# Intelligent blood collection robot: key technologies, clinical applications, and future challenges

**DOI:** 10.3389/fbioe.2025.1587114

**Published:** 2025-05-30

**Authors:** Yaqing Wang, Huajiang Ruan, Yaoqin Ruan, Qi Li, Chen Wang, Zhaoxi Fang

**Affiliations:** ^1^ Department of Transfusion Medicine, The Affiliated Hospital of Shaoxing University (Shaoxing Municipal Hospital), Shaoxing, China; ^2^ Department of Computer Science and Engineering, Shaoxing University, Shaoxing, China; ^3^ Institute of Artificial Intelligence, Shaoxing University, Shaoxing, China

**Keywords:** intelligent blood collection robot, vascular recognition, machine vision, puncture control, clinical application

## Abstract

Blood collection is one of the key steps in blood testing. The use of intelligent blood collection robots to achieve automated blood collection can effectively reduce the workload of medical staff, improve the efficiency and accuracy of blood collection, and improve the patient experience, and has received extensive attention from academic and industrial circles in recent years. This paper aims to provide a comprehensive introduction to the key technologies, latest research progress, current clinical application status, and future development opportunities and challenges of intelligent blood collection robots. The paper introduces the key technologies that can achieve autonomous blood collection, including blood vessel recognition and positioning technology, mechanical arm design, and puncture control technology. Among them, blood vessel recognition and positioning is one of the key supporting technologies for intelligent blood collection, which can be realized by using technologies such as machine vision, near-infrared imaging, and ultrasound imaging. In addition, the paper also introduces some existing commercial intelligent blood collection robots and their clinical application cases in hospitals. Finally, we analyze the future application prospects and challenges of intelligent blood collection robots.

## 1 Introduction

Blood testing, as a core component of medical laboratory diagnosis, plays a crucial role in disease prevention, diagnosis, treatment monitoring, and health assessment. Blood contains various cells and chemical components. Many diseases, such as infections, anemia, coagulation disorders, diabetes, kidney diseases, and liver diseases, can be identified by abnormal specific indicators in blood tests (Beili et al., 2021; Organization, 2010).

Blood collection is the primary step in blood testing, and its accuracy and reliability have a decisive influence on the subsequent test results. At present, the blood test analysis has been fully automated, but the blood sample collection is still completed manually. Manual blood collection has several problems, including slow blood collection, high failure rate, risk of infection, and long training time for blood collection skills. According to (Cheng, 2015), the failure rate of venous blood collection in a hospital medical examination center is 3%. In the process of blood collection, injuries often occur due to nurses’ non-compliance with protocols or misuse. H. Li et al. found that only 61.01% of nurses insisted on wearing gloves during blood collection, and 20.78% of needle-stick injuries occurred due to blood collection (Li et al., 2017). A survey in (Dulon et al., 2020) also showed that blood collection-related needlestick injuries accounted for more than 20%, which is one of the most prone to needlestick injuries in medical behavior.

Qualified blood drawing skills require long-term training and practice accumulation, which is a larger investment for medical institutions. Therefore, developing intelligent blood-drawing robots based on the latest generation of information and control technology to replace manual blood drawing is the future trend. Intelligent blood drawing robots have many advantages, including improving blood drawing efficiency and accuracy, reducing the waiting time for patients, reducing the risk of infection, improving the patient experience, reducing the workload of medical staff, and promoting the construction of medical informatization (Dong, 2019; Zheng, 2022).

The development of smart blood drawing robots involves multiple technologies, including machine vision technology, image fusion navigation technology, force feedback control technology, biometric identification technology, and multi-degree-of-freedom automatic puncture technology, which has a high degree of complexity (Liu, 2020; Wu, 2022; Xue Yang, 2020; Chen Z. et al., 2020; Liu, 2023). This paper aims to comprehensively sort out and summarize the latest research progress in the field of intelligent blood collection robots. This paper first describes the basic components of intelligent blood collection robots, and the different types of robots according to the site of blood collection, such as fingertip blood collection, vein blood collection, and blood collection from other body parts. Next, we analyze the key technologies and methods involved in intelligent blood collection robots, including machine vision technology, biometrics technology, infrared imaging technology, ultrasound imaging technology, and other crucial technologies. In Section 4, we investigate the current status of research and development and clinical application of existing blood collection robots and analyze the challenges and development opportunities that need to be addressed in the future in Section 5.

## 2 Types of intelligent blood collection robots

**FIGURE 1:**
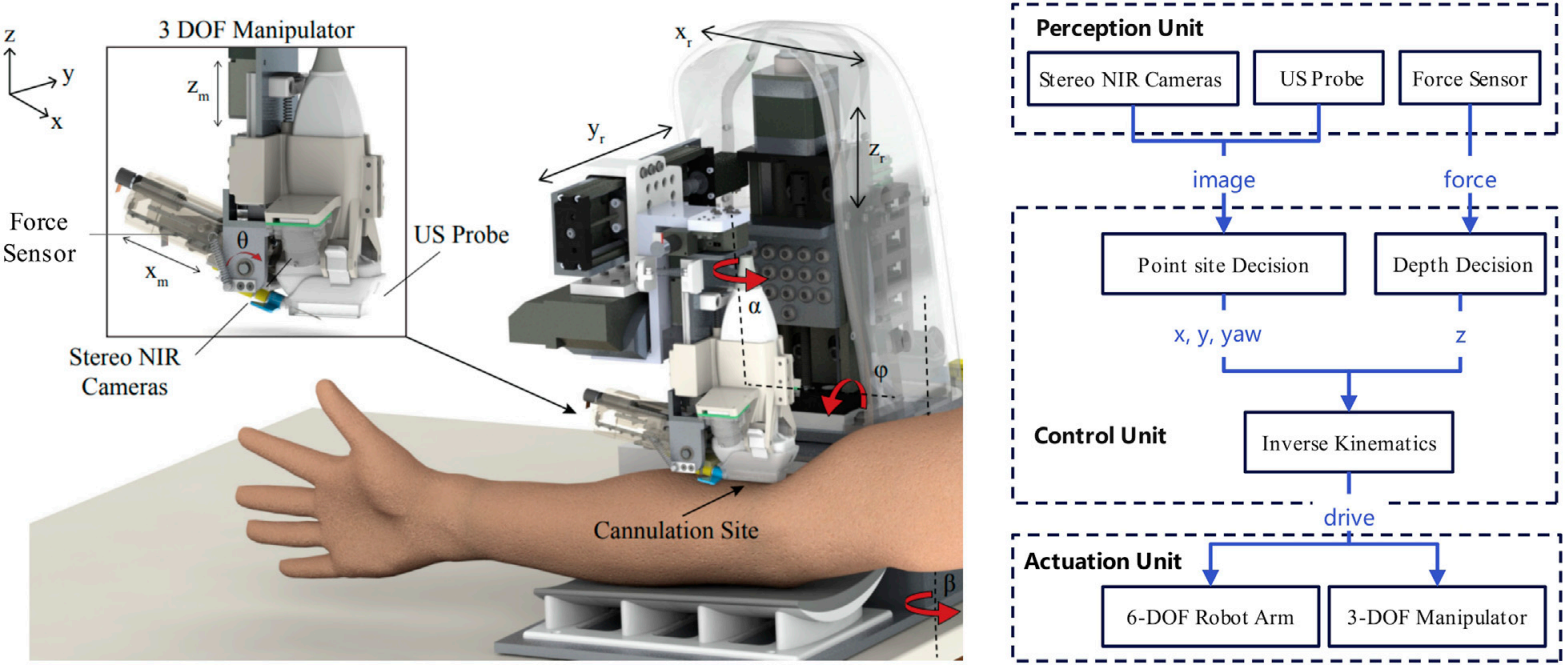
Structure of a US and NIR-Based robotic system for venous blood collection (Chen et al., 2015).

Figure 1 shows the structure of a typical blood collection robot. Among them, the robotic arm is responsible for performing specific blood collection operations and is one of the core parts of the robot. Sensors are used to obtain relevant information about the patient’s body, such as the position and depth of the blood vessels. Near-infrared (NIR) imaging cameras can penetrate the skin to accurately identify and restore the location of veins hidden under the skin, which is crucial for successful blood collection. Force sensors are used to monitor the force of needle insertion to avoid unnecessary injury to the patient. The control system acts as the brain of the blood collection robot, processing the data collected by the sensors, making decisions, and controlling the movement of the robotic arm. This often involves complex algorithms, including but not limited to deep learning and image processing technologies, to ensure accuracy and safety.

Depending on where the blood is collected, intelligent blood collection robots can be categorized as fingertip, upper extremity vein, and other sites. Below we will detail the characteristics of intelligent robots that collect blood at different sites and related research.

### 2.1 Fingertip blood collection robot

Fingertip blood collection robots are emerging medical devices that aim to automate the blood collection process and improve the efficiency and accuracy of blood testing, especially for diabetic patients who need to monitor their blood glucose on a regular basis. Fingertip blood collection robots are usually composed of multiple modules, including blood collection actuation module, information acquisition module and robotic arm system (Li et al., 2021c). The design of a fingertip blood collection robot relies on advanced vision systems, including near-infrared imaging and binocular vision technologies. These technologies enable the robot to effectively recognize finger veins and select the optimal blood collection point (Li et al., 2021b). By using a combination of 3D reconstruction and contour extraction, the robot is able to quickly acquire information about the location of the fingertip blood sample collection and perform precise puncture operations using laser guidance. This design gives the robot a significant advantage in terms of fast response and high-precision localization.

### 2.2 Upper extremity venous blood collection robot

Upper extremity venous blood collection is one of the most common operations in clinical diagnosis and treatment. As traditional manual blood collection relies on the experience and skills of medical personnel, the success rate of puncture for patients with obscure veins (e.g., infants, the elderly, and obese patients) is low, resulting in patient pain and waste of medical resources. Currently the main research hotspot is how to reduce the damage to blood vessels and improve the success rate of blood collection through the optimisation of robotic arms, needles, sensors and algorithms (Kobayashi et al., 2013; Yang et al., 2024b; Balter et al., 2016b; Yang et al., 2018).

### 2.3 Blood collection from other parts

In addition to fingertip and upper extremity vein blood collection, blood can also be collected from other parts of the body depending on the patient’s physical condition or disease treatment needs. For example, for newborns and infants, the heel of the foot is a common site for blood collection because of the thicker skin tissue in this area, which prevents damage to the nerves and blood vessels of the plantar foot. For special patients who are unable to find a suitable puncture vein in the limb, a superficial neck vein may be chosen for blood collection. In some special cases, such as severe burn patients, extremely obese patients, etc., the femoral vein may be selected for blood collection. In addition, arteries at the wrist are commonly used for arterial blood collection due to their relative superficiality and ease of collection (Ma et al., 2021; Brattain et al., 2021).

## 3 Key technologies for intelligent blood collection robots

Intelligent blood collection robots involve a number of key technologies, including advanced image processing and machine vision technologies, high-precision robotic arm design and control technologies, powerful data processing and decision-making systems, and comprehensive safety measures, which work together to ensure that the robot can efficiently, accurately and safely complete the venous blood collection task. Table 1 lists representative papers in the field of intelligent blood collection robotics. It gives the blood collection site, blood vessel recognition technique, target user and performance results of the robot studied in each paper.

**TABLE 1:** Blood collection robots in the literature.

References	Blood collection site	Vascular recognition technology	Other key technologies	Target user	Performance results
Li et al. (2021c)	Fingertips	Binocular vision and NIR	Laser guidance	General patient	Maximum deviation not more than 0.15mm; Positioning time: <9.8 s
Ma et al. (2021)	Upper limb veins	RGB-D cameras and US	PID force control; 3D trajectory planning; Active Contour	General patient	Positioning error: 0.3mm; success rate: 98%
Li et al. (2021a)	Fingertips	Binocular vision	3D reconstruction; Laser positioning	Hyperglycaemic patients	Tolerance <2mm; Time 1.8s
He et al. (2023a)	Upper limb veins	NIR	SAU-Net; Position and attitude adjustment	Patients with complex vascular conditions	Segmentation accuracy 91.2%; Decision time 1.458 s
He et al. (2023b)	Upper limb veins	NIR	DL; CNN-based semantic segmentation	General patient	High accuracy of elbow vein recognition
Wang et al. (2023b)	Upper limb veins	NIR	YOLOv5; U-Net	Adults and Children	Detection accuracy: 99.3%; Segmentation accuracy: 89.1%; average test time: 0.55s
Long et al. (2022)	Upper limb veins	NIR	DL; U-Net	Patients with low visible veins	N/A
Salcedo and Peñaloza (2023)	Upper limb veins	NIR	Laser sensor; Force feedback	Adults and Children	Positioning accuracy 0.11mm; Repeatability error 0.04 mm
Leipheimer et al. (2019)	Upper limb veins	US	Microbot	Adults	Overall success rate: 87%; Success rate in non-DVA patients: 97%
Ning et al. (2023)	Upper limb veins	US	DL; RL; Force control	Adults	N/A
Dong et al. (2020)	Upper limb veins	US	Faster-RCNN; Automatic threshold segmentation	General patient	N/A
Wu et al. (2024)	Vein	US	UV-YOLOv7; DN-DBSCAN	General patient	Inference time: 0.6 m
Watanabe et al. (2024)	Peripheral vein	US	U-Net; Hough transform	Adults and Children	N/A
Wang et al. (2023a)	Upper limb veins	Camera and NIR	Image navigation control technology	General patient	Puncture success rate 94.3%
Chen et al. (2020a)	Upper limb veins	NIR and US	DL; Robot servo control	General patient	Puncture success rate 88.2%
Kim et al. (2024)	Vein	NIR and US	Automatic needle exchange system; PID control	General patient	Success rate of 3–5 mm endovascular puncture: 97.3%
Ji et al. (2022)	Vein	NIR and US	Mixer-UNet; MLP-Mixer	30 volunteers	Accuracy: 93.07%, F1 score: 78.37%
Zhang et al. (2023)	Upper limb veins	NIR and US	DDPG; Robotic arm motion planning	General patient	Success rate over 95%; Positioning error: 1.55 mm
Balter et al. (2016a)	Upper limb veins	NIR and US	Force feedback; Image guidance; Microfluidic analysis	Children, elderly, obese patients	Nearly 100% puncture success rate, with an error of less than 0.3 mm
Cao et al. (2021)	Upper limb veins	NIR and US	Automated positioning algorithms	Adults	Average DSC 0.7634; Angular error 15.58°
Lin et al. (2022)	Upper limb veins	NIR and US	DL; Vision real-time tracking; Laser sensor; Force feedback	Adults; Difficult patients	Success rate: 90%; Positioning accuracy: 0.05 mm
Sha et al. (2022)	Upper limb veins	NIR and US	Pressure sensor; YOLO-V5; motion control algorithm	Adults; Tumour patients	Success rate 91%; Positioning error 0.05 mm
Biocheng Group (2024)	Upper limb veins	NIR and US	Six-dimensional force sensing; Nine-axis flexible control	Children; The elderly	Animal testing success rate: 95%

### 3.1 Vessel identification and localization

#### 3.1.1 Camera-based vessel identification and localization

Camera-based machine vision technology is a computer technology that simulates the human visual system to recognize and understand objects through image acquisition, processing, and analysis. In the intelligent blood collection robot, this technology is used to achieve accurate positioning and identification of veins. Through a high-resolution camera combined with advanced image processing algorithms, the robot is able to analyze the subtle features of the skin surface in real-time, identify the morphology, location, and depth of the vein, and intelligently plan the navigation of the puncture path (Hu et al., 2023). The application of this technology not only improves the success rate of blood collection, but also significantly reduces the invasiveness to the patient and enhances the overall medical experience.

In terms of robot path planning and navigation, Li et al. in (Li et al., 2021a) developed a binocular vision-based robot navigation system for fingertip blood collection. The system obtains the coordinates of the fingertip blood collection point through 3D reconstruction and contour extraction, and optimizes the scale-invariant feature transform (SIFT) operator to improve the efficiency and stability of feature point description. The experimental validation results show that the method can quickly and accurately guide the robotic arm to move the blood collection needle to the target position, which effectively meets the navigation needs of the blood collection robot.

#### 3.1.2 NIR-based vessel identification and localization

Infrared imaging technology is playing an increasingly important role in modern healthcare, especially in the automation of venipuncture and fingertip blood collection. Based on the reflective properties of hemoglobin to near-infrared (NIR) light, this technology can efficiently acquire venous image data to help medical robots localize blood vessels and perform precise manipulations.

The venipuncture robot developed by He et al. (He et al., 2023a) achieved accurate segmentation of vein images by combining near-infrared imaging with the SAU-Net model, with a segmentation accuracy of 91.2%, while the vein extraction rate was 86.7%. The average decision time of the system was 1.458 s. The embedded elbow vein blood collection robot studied by Wang et al. (Wang H. et al., 2023) combined NIR spectroscopy technology with deep learning algorithms to achieve high-precision recognition of elbow veins, which reasonably reduced the duplication of labor by healthcare workers and significantly improved the efficiency of blood collection. Meanwhile, Long et al. (Long et al., 2022) developed an automatic elbow vein puncture point detection method based on YOLOv5 and U-Net, which significantly improved the puncture efficiency and accuracy through efficient segmentation and precise localization, and the detection accuracy reached 0.993, which meets the clinical real-time demand.

#### 3.1.3 Ultrasound-based vessel identification and localization

Ultrasound imaging plays a key role in interventional medicine, by using high-frequency sound waves to obtain three-dimensional coordinates of blood vessels, including important parameters such as diameter, depth, and vessel wall characteristics. Leipheimer et al. (Leipheimer et al., 2019) developed a handheld automated blood collection device that uses ultrasound imaging to identify vessels suitable for puncture and robotics to precisely guide the needle into the center of the vessel. This innovative device performed well in clinical trials with an overall success rate of 87% and a success rate of 97% in non-difficult venous participants, demonstrating its potential to significantly improve the efficiency and safety of blood collection. The authors in (Ning et al., 2023) proposed a novel approach that combines a weakly supervised ultrasound vascular segmentation network with a reinforcement learning strategy. This enabled the system to perform stable vascular imaging on different human surfaces without additional tuning. Meanwhile, Dong et al. (Dong et al., 2020) utilized deep learning and the Faster-RCNN algorithm to achieve coarse position recognition of blood vessels in ultrasound images and accurately measure the vessel positions and diameters by an automatic threshold segmentation algorithm. Such technological breakthroughs improved the repeatability and accuracy of the robot in venipuncture experiments.

The ultrasound-guided venipuncture vessel identification system proposed by Wu et al. (Wu et al., 2024) optimized the processing of blood vessels in ultrasound images by the K-means clustering method and designed a lightweight UV-YOLOv7 network to significantly improve the processing speed and accuracy of the model. The DN-DBSCAN algorithm introduced by the system effectively eliminates the misdetection region, resulting in a vessel localization accuracy of mAP 86.2%. Meanwhile, H. Watanabe et al. (Watanabe et al., 2024) used the U-Net convolutional neural network and traditional image processing techniques to achieve high-precision segmentation of blood vessels and puncture needles.

#### 3.1.4 The integration of multiple technologies

Multimodal sensing technology realizes high-precision blood vessel localization and puncture operation by integrating multiple sensors such as near-infrared, ultrasound, and vision, and combining them with AI data fusion algorithms, which shows great potential in intelligent blood collection robot systems. The synergy of different sensors can make up for the limitations of a single sensor and provide reliable technical support for automated operations in complex medical environments.

##### 3.1.4.1 Combination of visible light and infrared

Wang et al. (Wang C. et al., 2023)developed an intelligent blood collection robot, which realized accurate identification of blood vessels and automated blood collection through artificial intelligence machine vision and image navigation control technology. The technology adapted to diverse blood vessel characteristics through big data analysis and excelled in controlling blood collection volume and reducing pain, with a puncture success rate of 94.3%. Although the robot’s collection results differed in 11 anticoagulant indicators compared to manual blood collection, this did not affect the reliability of clinical diagnosis.

In terms of fingertip blood collection, the intelligent fingertip blood collection robot of Li et al. (Li et al., 2021c) optimized vein recognition by combining near-infrared imaging technology with a binocular vision system (Li et al., 2021a), and was able to increase the bleeding volume by automatically selecting the area of vein intersection as the blood collection point. The experimental results show that the maximum deviation between the needle and the blood collection point of this robot does not exceed 0.15 mm, and the blood collection time is less than 9.8 s, demonstrating the potential application of NIR imaging in fingertip blood collection scenarios.However, this combination also has limitations. Visible light imaging can be affected by skin color and lighting conditions, while infrared imaging may struggle with accurately determining the depth of blood vessels. Additionally, the integration of these two imaging modalities requires sophisticated algorithms and hardware to ensure real-time processing and accurate fusion of the images. Despite these challenges, the combination of visible light and infrared imaging offers a promising solution for improving the performance of intelligent blood collection robots.

##### 3.1.4.2 Combination of visible light and ultrasound

The combination of ultrasound sensing and machine vision provides an efficient solution for vessel localization in complex medical scenarios. Ma et al. (Ma et al., 2021) developed a robotic system integrating an RGBD camera and a 2D ultrasound sensor to achieve safe scanning and accurate localization through a PID force controller and real-time calibration. The system was shown to have a positioning deviation of only 0.3 mm, which is close to the theoretical resolution of clinical ultrasound systems, and has built-in safety measures to support future human testing. The system utilizes surface point clouds to generate automated scanning trajectories, which are combined with real-time analysis of ultrasound images to successfully reconstruct blood vessels in three dimensions. This multimodal fusion technology significantly improves the accuracy and safety of vascular localization, laying the foundation for clinical applications.

Despite its advantages, the combination of visible light and ultrasound also presents challenges. Ultrasound imaging requires close contact with the skin and the use of coupling agents, which may introduce additional complexity and potential hygiene issues. Moreover, the interpretation of ultrasound images requires specialized knowledge and experience, and the quality of the images can be influenced by factors such as the patient’s body type and the presence of air pockets. These factors may affect the accuracy and reliability of blood vessel localization.

##### 3.1.4.3 Combination of infrared and ultrasound

The combination of NIR and ultrasound is particularly common in vascular localization, with the advantage of utilizing NIR to achieve surface distribution detection of blood vessels while acquiring depth information through ultrasound. The authors in (Chen A. I. et al., 2020) developed a portable robotic system combining NIR and US imaging to achieve high-precision segmentation and depth estimation of blood vessels through deep learning, which demonstrated significantly better performance than manual operation in simulation and *in vivo* experiments, verifying its efficiency and clinical application value.

The Intelligent Venous Injection Robot (IVIR) developed by Kim et al. (Kim et al., 2024) uses NIR and US imaging to improve the accuracy of venipuncture. The system uses NIR imaging to determine the 2D position of the vessel and estimates the vessel depth through a U-net network for accurate 3D localization. In a simulated environment, the IVIR system achieved a puncture success rate of 97.3%, demonstrating high accuracy and reproducibility for vessels with diameters of 3 mm and above. Although this result originated in the field of injections, the precise localization technique is also applicable to the blood collection process. The Mixer-UNet model proposed by Ji et al. (Ji et al., 2022), on the other hand, significantly optimizes the fusion performance of NIR and US imaging in vein segmentation and 3D localization by combining UNet with MLP-Mixer structure. The model performed excellently in the segmentation task, with test results showing a segmentation accuracy of 93.% and an F1 score of 78.37%.

However, the integration of NIR and ultrasound imaging also has its limitations. NIR imaging may be affected by factors such as skin pigmentation and the presence of hair, which can interfere with the detection of blood vessels. Ultrasound imaging, as mentioned earlier, requires proper contact with the skin and may be sensitive to movements, which can lead to image distortion and affect the accuracy of depth estimation. Furthermore, the integration of these two imaging modalities demands advanced data fusion algorithms and computational resources to process and fuse the images in real time.

##### 3.1.4.4 Comparison of different sensor combinations

Each sensor combination has its unique advantages and limitations, making them suitable for different clinical scenarios and patient populations. The combination of visible light and infrared imaging is ideal for situations where surface and subsurface blood vessel information is crucial, such as in patients with visible veins. It offers high-resolution imaging and the ability to detect veins beneath the skin, but may be less effective in determining the exact depth of the vessels. On the other hand, the combination of visible light and ultrasound provides detailed 3D information about the blood vessels, including their depth and diameter, making it suitable for more complex cases where precise localization is essential. However, it requires more specialized hardware and expertise to operate effectively. The integration of infrared and ultrasound imaging strikes a balance between surface and depth detection, offering a comprehensive view of the blood vessels. This combination is particularly useful in cases where both the surface distribution and depth of the vessels are critical for successful puncture. However, it also demands sophisticated algorithms for data fusion and interpretation, and may be more sensitive to external factors such as skin condition and patient movement. In conclusion, the choice of sensor combination depends on the specific requirements of the clinical application, the characteristics of the patient population, and the available technological infrastructure. Future research in this area should focus on developing more advanced data fusion algorithms and hardware systems that can maximize the strengths of each sensor while minimizing their limitations. This will enable intelligent blood collection robots to perform more accurately and reliably across a wider range of clinical scenarios.

### 3.2 Robotic arm design

**FIGURE 2:**
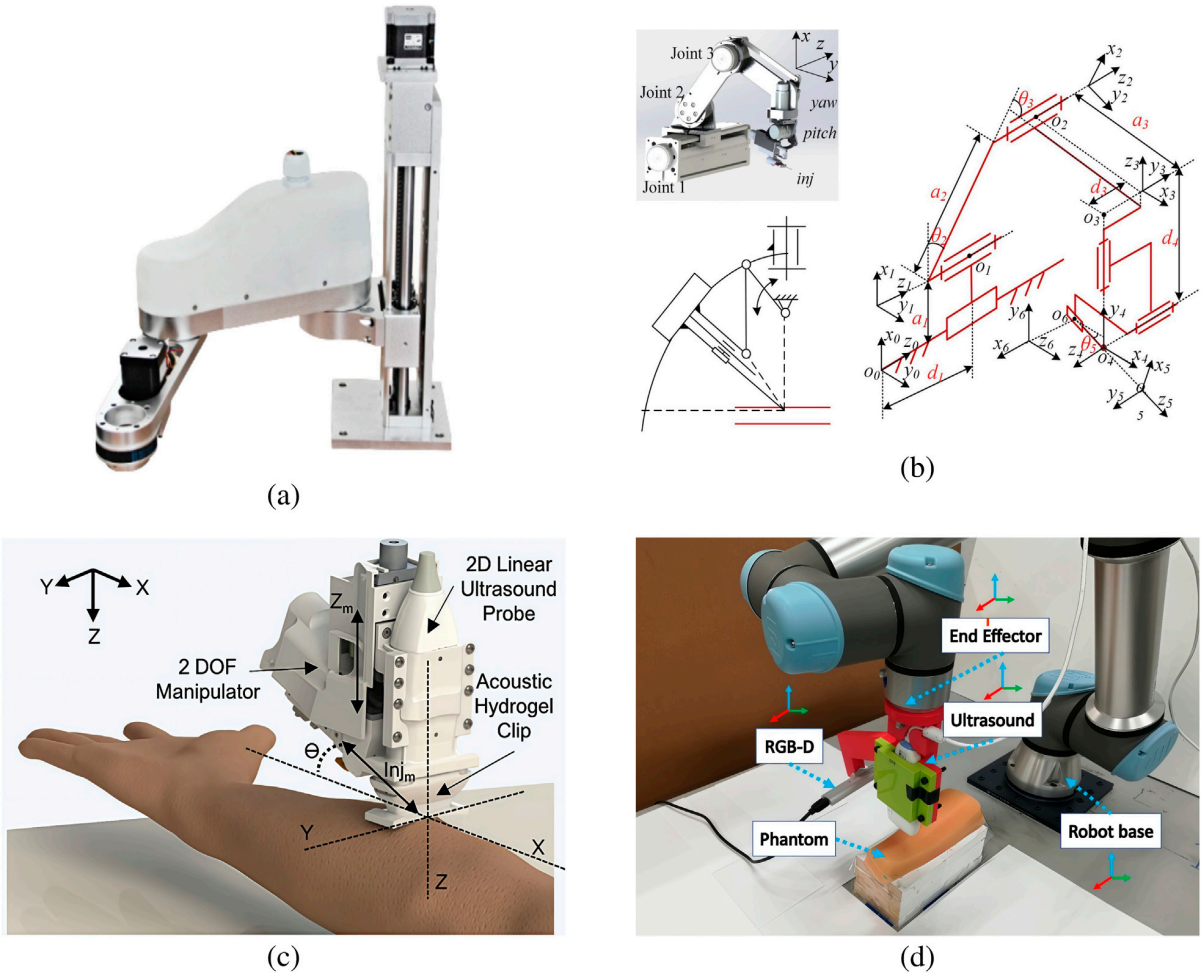
Several vascular puncture module designs. **(a)** Ultrasound-based puncture structure (Dong et al., 2020). **(b)** Camera and NIR-based puncture module design (Li et al., 2021b). **(c)** 3D model of the puncture device (Yang et al., 2024a). **(d)** Infrared-based puncture module (He et al., 2023a).

In the design of venipuncture robots, the robotic arm, as a key moving component, is not only crucial for improving puncture accuracy, but also significantly reduces the patient’s discomfort experience through the optimized design of structure and control, as shown in Figure 2. Balter et al. Balter et al. (2016b) designed a 9-DOF venipuncture robot with integrated NIR and US imaging. The device achieves precise positioning through computer vision and force feedback, and the optimized mechanical configuration, integrated design of the imaging and puncture subsystems, and the addition of force sensors allow for sub-millimeter positioning accuracy and repeatability in the operational workspace.

Yang et al. (Yang et al., 2024a) proposed a blood collection robot based on a rigid-flexible composite puncture strategy. The robot combines a 7-degree-of-freedom robotic arm with a cannulated needle design to avoid damage to the lower wall of the blood vessel during puncture. The feasibility of its composite puncture strategy and the accuracy of its trajectory planning are experimentally verified by solving the inverse kinematics using a differential evolutionary algorithm and trajectory planning using fifth-degree polynomial interpolation. This innovative strategy significantly improves puncture accuracy and provides technical support for reducing patient pain.

In addition, He et al. (He et al., 2023b) developed a 6-degree-of-freedom venepuncture robot with decoupled position and attitude control via near-infrared vision and force feedback. It adopts an improved double-parallel four-link structure, which makes the overall design more compact and the motion pattern concise and efficient. The needle tip position remains stable during the needle attitude adjustment process, which significantly improves the puncture accuracy and simplifies the operation control, demonstrating the advanced technology of the venipuncture robot and laying a solid foundation for its wide application in the field of intelligent medical treatment.

### 3.3 Puncture control

In addition to the precise positioning of blood vessels, the role of high-precision motion control systems in the blood collection process is crucial to effectively improve puncture accuracy and reduce patient pain. During venipuncture, biometric technology monitors the impedance changes of tissues in real-time through electrical impedance measurements, which can accurately differentiate between tissues such as blood vessels, fat and muscle. Specifically, the tip of the needle is equipped with an electrode sensor that applies a weak current and analyzes the feedback voltage signal to calculate conductivity. These data are not only used to determine the relative position of the needle tip to the tissues but also to construct a biomechanical model to simulate the interaction between the needle tip and the surrounding tissues to optimize the puncture path (Cheng et al., 2016). In addition, by combining the precise positioning and control system of the robot, this technique can automatically adjust the depth during the puncture process, which significantly improves the success rate and safety of the puncture.

To cope with possible complications during venipuncture, Liu et al. (Liu et al., 2021) proposed a method based on electrical impedance measurement. They used agar and gelatin to make tissue phantoms simulating blood, fat and skin, and measured the electrical conductivity of the phantoms by means of homemade electrode probes and sodium chloride solutions of different concentrations. The results showed that the conductivity of the fabricated phantom matched well with the real tissue data. In addition, Smirnova et al. (Smirnova et al., 2023) developed an automated vein visualization module based on bioimpedance measurements designed for intelligent blood collection robots. The module accurately recognizes the location of the vein by means of an array of electrodes, which guarantees an accuracy rate of over 90% for the puncture control unit. Compared to traditional ultrasound and optical imaging systems, this technology interferes less with patient movement and is particularly suitable for special groups such as pediatrics, enhancing the safety and comfort of the medical process.

## 4 Clinical applications

**FIGURE 3:**
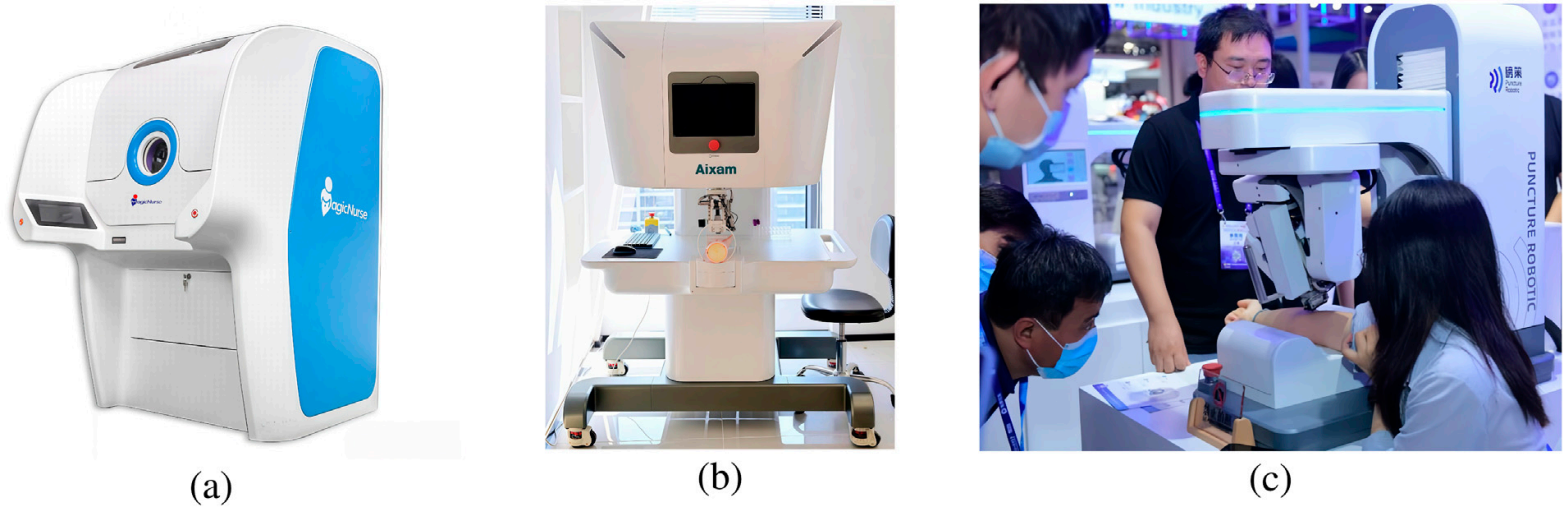
Several representative blood collection robots. **(a)** MagicNurse (Artery Network, 2023). **(b)** Aixam (Biocheng Group, 2024). **(c)** Hagong (Sohu News, 2023).

So far, a number of companies have launched commercial blood collection robots and have clinical applications in some of China’s leading hospitals, as shown in Figure 3.

### 4.1 Commercially available intelligent blood collection robots

MagicNurse intelligent blood collection robot is an AI medical robot that can realize unmanned and standardized blood collection in the whole chain, and can replace nurses to collect venous blood automatically and unattended (Artery Network, 2023). By recruiting volunteers from the society, as of January 2021, MagicNurse robot has carried out more than 3,000 cases of real-life puncture blood collection tests, and the accuracy of the first needle puncture has reached about 95%.

In 2024, Kerry Medical released an optical-acoustic all-in-one mechanic’s multimodal intelligent perception puncture robot, the Aixam Divine Needle intelligent blood collection robot (Biocheng Group, 2024). The robot has completed animal experiments on a third-party experimental platform: blood collection from the tail vein of white rats, and the success rate of blood collection reaches 95%. In the animal experiments, the diameter of the puncture blood vessel ranges from 0.44 to 0.8 mm, with an average diameter of 0.61 mm, and the experimental blood collection needle diameter is 0.45 mm. The diameter of the experimental blood collection needle was 0.45 mm.

The Hagong blood collection robot (Sohu News, 2023) is equipped with body fluid and blood collection analysis, temperature monitoring, 5G remote diagnosis, automatic disinfection of negative pressure, etc. The device incorporates advanced technologies, including AI reconstruction recognition, image fusion navigation, and force feedback control. It is the inaugural system to offer real-time image analysis and rapid calculation of tissue morphology. The robot is capable of adopting CT and ultrasound image fusion alignment navigation, real-time feedback of soft tissue changes, ultrasound posture fitting, and solving the standard stay map.

On 30 May 2022, Vitestro presented the company’s development of an autonomous blood-drawing device (Vitestro, 2023). Vitestro’s device combines artificial intelligence-based ultrasound-guided 3D reconstruction with robotic control technology to ensure accurate and safe blood collection. In clinical studies, Vitestro has performed 1,500 automated blood draws on more than 1,000 patients using its prototype. A single healthcare professional can oversee multiple devices and manage multiple patients at the same time, thus alleviating the shortage of blood collection nurses in hospitals.

### 4.2 Clinical application cases of blood collection robot

On 17 September 2021, an intelligent blood collection robot was officially deployed in Sun Yat-sen Hospital of Fudan University (Government Affairs, 2023). This is the inaugural intelligent blood collection robot to be installed in Shanghai. As detailed in the paper published by the hospital’s doctors in 2023 (Wang C. et al., 2023), 154 volunteers from Sun Yat-sen Hospital of Fudan University were recruited over the 2-year period during which the robot was deployed. The results of anticoagulant blood samples were compared between the robotic and manual blood collection methods, and a questionnaire was used to ascertain the volunteers’ perceptions of the two methods of blood collection. Furthermore, the study gathered data pertaining to the utilization of robotic blood collection from 6,255 patients who had consented to this method of blood collection, and proceeded to analyze the success rate of said collection. The findings of the study indicated that the blood collection robot demonstrated superior performance compared to manual collection in terms of specimen volume, pain, and puncture success rate, with a puncture success rate of 94.3%. A total of 11 indicators demonstrated statistically significant differences between the anticoagulated blood specimens collected via robotics and those obtained through manual methods. However, these discrepancies did not impact the clinical diagnosis or prognosis.

On 3 June 2021, the first automatic blood collection robot in the province was officially put into use in the Fourth Hospital Affiliated to Zhejiang University School of Medicine (DXY, 2023). On that morning, the self-service blood collection robot successfully completed the blood collection service for over ten patients, achieving a 100% success rate.

In 2023, the First Hospital of Anhui Medical University established a digital intelligent blood collection facility at Jixi Road Hospital, comprising three MagicNurse intelligent puncture blood collection robots (Tang et al., 2023). From the second half of 2023 to May 2024, the aforementioned digital intelligent blood collection area has successfully completed blood collection for more than 5,100 patients. The automatic blood collection machine is capable of continuous operation, facilitating the completion of blood collection tasks at any given moment. Its functionality encompasses enhanced speed and success rates in blood collection, effectively transitioning from manual blind puncture to precise visual automatic puncture. This approach is particularly beneficial for individuals experiencing dizziness or discomfort during the blood collection process, while also mitigating the risk of potential complications.

## 5 Discussion

Despite encouraging progress, a number of challenges remain in the widespread adoption and optimization of artificially intelligent blood collection robots. A major challenge is ensuring the reliability and consistency of the technology across different patient populations. Variations in skin color, vein visibility and patient movement can affect robot performance, so there is a need to continually improve vessel recognition and positioning accuracy. Another challenge is the need to improve the safety and reliability of the device to ensure system stability under prolonged, high-frequency use. In addition, extensive training and education of healthcare providers is needed to effectively integrate these robots into clinical workflows.

The accurate location of blood vessels represents a fundamental challenge for the development of intelligent blood collection robots capable of automated puncture. Despite notable advances in vein recognition technology, including machine vision, near-infrared imaging, and ultrasound imaging, as well as other techniques, the complexities of clinical environments, such as light interference, the diversity of skin features, the depth of vessel location, and the variability of anatomical structures, continue to challenge the robustness of the algorithms employed. Furthermore, the potential for patients to experience changes in their physiological state due to external factors such as tension or movement serves to compound the challenge of accurate localization. It is therefore imperative that future research incorporates multimodal sensor data fusion techniques and introduces real-time adaptive learning models, thereby enabling the robot to dynamically capture changes in vascular features and achieve high-precision continuous tracking.

The conventional methodologies employed for the control and operation of robotic arms are inadequate for fulfilling the rigorous demands for flexibility in venipuncture procedures. It is essential that intelligent blood collection robots are able to respond rapidly to minor patient movements while simultaneously maintaining high-precision path planning of the robotic arm within three-dimensional space. This necessitates not only the optimization of the redundant degrees of freedom of the robotic arm, but also the enhancement of the force feedback sensing system, which enables it to precisely adjust the depth and angle of needle insertion in milliseconds, thus avoiding unnecessary damage to the surrounding tissues (Cheng et al., 2016). The future development of puncture operating systems will be focused on the creation of lighter, more sensitive models through the combination of flexible actuators with biomechanical modelling, achieved through the optimization of both hardware and software.

It is possible that sensors, actuators and control algorithms may experience a decline in performance over time when subjected to high-frequency use. Furthermore, the repeatability and positioning accuracy of the robotic arm may diminish over time. It is therefore essential that intelligent blood collection robots are equipped with efficient self-calibration and health monitoring modules that are capable of assessing the status of core components in real time. This will enable the early detection of any issues and the implementation of necessary adjustments to ensure that the equipment is always operating at optimal performance, thereby preventing accidents.

## 6 Conclusion

Intelligent blood collection robots represent a significant advancement in the field of medical automation. These devices are equipped with advanced capabilities, including autonomous vein detection and precise blood collection, which are enabled by machine learning, image recognition, and automatic control technologies. These technologies are poised to revolutionize the way blood is collected, potentially replacing traditional manual methods. Theoretical studies and clinical experience have demonstrated that intelligent blood collection robots have the potential to enhance the efficiency, accuracy and comfort of blood collection.

However, they also face challenges pertaining to technology, market acceptance, and cost. Currently, the market acceptance of robotic blood collection is relatively low, with a limited number of clinical applications in both domestic and international settings. Consequently, the market is currently in a state of cautious observation. Nevertheless, in light of the national focus on medical automation, it is anticipated that there will be an increase in the number of policies designed to facilitate the advancement and implementation of intelligent blood collection robots. The potential applications of intelligent blood collection robots are numerous and diverse. In addition to their suitability for use in hospitals, they can also be deployed in clinics, mobile blood collection vehicles, physical examination centers and other scenarios. It is anticipated that, in the future, as technology progresses and market demand increases, intelligent blood collection robots will become more widely applicable.
